# Body roundness index and its association with QUS-defined osteoporosis and bone mineral density in middle-aged and older adults: a cross-sectional analysis

**DOI:** 10.3389/fendo.2026.1906809

**Published:** 2026-08-12

**Authors:** Yucong Yang, Qichao Wang, Xiuwei Hou, Zhiwei Huang, Jing Bai, Bing He, Li Wu, Li Guo, Jirui Cai, Jin Wang, Qiaotao Xie, Haoran Wang

**Affiliations:** 1 Henan University College of Medicine, Henan University, Kaifeng, China; 2 Luohe Central Hospital, Luohe Medical College, Luohe, China; 3 The Second Affiliated Hospital of Luohe Medical College, Luohe, China

**Keywords:** Body Roundness Index (BRI), cross-sectional study, optimal cutoff, osteoporosis, risk stratification

## Abstract

**Objectives:**

This study evaluated the association between Body Roundness Index (BRI) and QUS-defined osteoporosis, its discriminative ability, empirical cutoff, and subgroup heterogeneity in middle-aged and older adults.

**Methods:**

We analyzed cross-sectional data from 1,444 community-dwelling adults aged 35–75 years. Osteoporosis was defined using tibial quantitative ultrasound-derived T-scores. Receiver operating characteristic curves assessed discrimination, restricted cubic splines examined dose-response relationships, logistic and robust regression analyses quantified associations, and subgroup analyses evaluated effect modification.

**Results:**

The Youden index identified an optimal BRI cutoff of 4.5. High BRI (≥ 4.5) was associated with higher odds of osteoporosis in the unadjusted model (OR = 1.606, 95% CI: 1.292–1.996; P < 0.001), the age- and sex-adjusted model (OR = 1.432, 95% CI: 1.141–1.797; P = 0.002), and the fully adjusted model excluding BMI (OR = 1.404, 95% CI: 1.105–1.783; P = 0.005). The association was approximately linear (P for non-linearity = 0.539; P-overall < 0.001). BRI alone showed modest discrimination (AUC = 0.562, 95% CI: 0.529–0.594), which increased after adjustment for age and sex (AUC = 0.680, 95% CI: 0.652–0.710) and after full covariate adjustment (AUC = 0.695, 95% CI: 0.674–0.731). The association was stronger among participants with metabolic syndrome (OR = 2.62, 95% CI: 1.641–4.179) than among those without it (OR = 1.39, 95% CI: 1.040–1.860; P for interaction = 0.022).

**Conclusions:**

Higher BRI was associated with QUS-defined osteoporosis, but BRI alone showed limited discrimination, and the fully adjusted model remained only moderately discriminative. BRI should therefore be considered an adjunctive anthropometric indicator within a broader assessment framework, rather than a stand-alone predictor or screening tool.

**Clinical trial registration:**

https://www.chictr.org.cn/showproj.html?proj=187762, identifier ChiCTR2300071492.

## 1 Introduction

Osteoporosis, a chronic metabolic bone disorder characterized by low bone mineral density (BMD) and microarchitectural deterioration, imposes a substantial global public health burden—affecting over 20% of adults aged ≥ 60 years and contributing to 8.9 million fragility fractures annually. These fractures reduce functional independence and increase 1-year mortality by 20–30%, making early risk stratification critical amid an aging population (1). Nutrition, overall dietary patterns, and body composition are important modulators of bone health, and accumulating evidence indicates that lifestyle-related factors and excess adiposity are closely linked to skeletal integrity (2–4). Traditional anthropometric metrics, such as body mass index (BMI) and waist circumference (WC), only partially capture the complexity of body composition (5, 6). BMI fails to distinguish fat from lean mass, while WC overlooks the three-dimensional distribution of visceral adiposity—a driver of metabolic dysfunction and low-grade inflammation (7, 8). The Body Roundness Index (BRI), derived from WC and height, has been examined in previous population studies of osteoporosis and related bone outcomes. However, its performance and association may vary across populations and bone-assessment methods (9–13).

Previous studies, including analyses of national survey data and other cohorts, have already examined BRI in relation to osteoporosis and bone outcomes (5–8, 11–13). Evidence remains limited, however, for community-based Chinese populations assessed with tibial quantitative ultrasound (QUS). Within this setting, direct comparisons of BRI with BMI and WC under the same adjustment framework and a cohort-specific empirical cutoff have not been well characterized (11–22). Potential heterogeneity by age, sex, and metabolic syndrome also remains incompletely characterized (7, 8). The present study therefore provides a regional and method-specific extension of existing evidence rather than proposing a new conceptual link between body shape and bone health.

Using baseline data from a community-based Chinese cohort, we evaluated the association between BRI and QUS-defined osteoporosis, characterized its dose–response pattern, derived an empirical cutoff, and compared its discriminative performance with BMI and WC. The analysis was motivated by prior evidence linking visceral adiposity to metabolic and inflammatory pathways relevant to bone health (20, 23–25). BRI can also be calculated from routine waist and height measurements (9, 10, 26), although ease of calculation does not establish clinical utility. We hypothesized that higher continuous BRI would be associated with higher odds of osteoporosis and that the association would be approximately linear. We also evaluated whether adding age and sex improved discrimination and explored heterogeneity across prespecified subgroups (7, 8, 23).

## 2 Materials and methods

### 2.1 Study design and population

This cross-sectional study was based on baseline data from the “Longitudinal Investigation of Osteoarthritis and Cardiovascular Health Status” cohort (27), a prospective observational study conducted in Luohe City and its surrounding areas, China, between November 2023 and November 2024. The study followed the Strengthening the Reporting of Observational Studies in Epidemiology (STROBE) guideline to ensure methodological rigor and transparent reporting (28).

Inclusion criteria were as follows: (1) middle-aged and older adults aged 35–75 years; (2) permanent local residency for ≥ 5 years to ensure follow-up feasibility; (3) willingness to undergo comprehensive assessments, including BMD measurement, echocardiography, and joint imaging; and (4) ability to complete long-term follow-up (≥ 5 years). Exclusion criteria included acute cardiovascular events (e.g., myocardial infarction, cardiogenic shock), malignant tumors, life-threatening trauma, severe psychiatric or cognitive impairment, and chronic kidney disease with an estimated glomerular filtration rate < 30 mL/min/1.73 m2. Of 1,762 participants initially assessed, 318 were excluded because of missing BRI (n = 1) or missing osteoporosis assessment (n = 317), leaving 1,444 participants in the primary analytic population. The same exposure/outcome complete-case dataset was used for baseline, regression Model 1, regression/ROC Model 2, restricted cubic spline (RCS), LOESS, distribution, and subgroup analyses; fully adjusted Model 3 analyses additionally required complete covariate data and included 1,408 participants.

### 2.2 Data collection

#### 2.2.1 Baseline characteristics

Comprehensive baseline data were collected through standardized questionnaires, physical examinations, and laboratory tests. Demographic variables included age and sex. Lifestyle factors included current smoking status (yes/no) and alcohol use (yes/no). Medical history of chronic conditions, including hypertension, diabetes mellitus, coronary heart disease (CHD), stroke, and metabolic syndrome (MS), was confirmed by self-report and medical records.

Biochemical markers were measured from fasting venous blood samples, including total cholesterol (TC), low-density lipoprotein cholesterol (LDL-C), high-density lipoprotein cholesterol (HDL-C), triglycerides (TG), and glycated hemoglobin A1c (HbA1c). Anthropometric measurements included height, weight, waist circumference, and BMI, calculated as weight (kg) divided by height squared (m²). Systolic blood pressure (SBP) and diastolic blood pressure (DBP) were measured in the sitting position using a calibrated sphygmomanometer, with the average of two consecutive measurements recorded.

#### 2.2.2 BRI measurement

BRI was measured using standardized anthropometric protocols, with values converted to a numeric format for analysis. BRI was calculated using the following formula: BRI = 364.2 − 365.5 × √(1 − (WC/(2π))²/(0.5 × height)²), where WC is waist circumference (cm), and height is measured in centimeters (cm). The consistency of BRI measurements was ensured through standardized training of research staff, and data were checked for completeness and accuracy before analysis.

#### 2.2.3 Osteoporosis and BMD-related measurements

BMD was assessed at the distal one-third of the left tibia using quantitative ultrasound (QUS) with the OsteoKJ7000+ device (Nanjing, China). For each participant, three consecutive Speed of Sound (SOS) measurements were obtained, and T-scores were automatically generated by the device’s built-in software using a manufacturer-specific reference database. BMD categories were defined as normal (T-score ≥ −1.0), osteopenia (−2.5 < T-score < −1.0), and osteoporosis (T-score ≤ −2.5). Although these T-score thresholds are analogous to the WHO diagnostic criteria for DXA-based osteoporosis, QUS measures bone quality (speed of sound) at a peripheral skeletal site rather than areal bone mineral density at the hip or spine, which is the clinical reference standard. Tibial QUS has moderate sensitivity and specificity for identifying DXA-defined osteoporosis (26), and QUS-based classification may introduce misclassification relative to DXA-based diagnosis. Accordingly, ‘osteoporosis’ in the present study denotes QUS-defined low bone quality throughout.

For the main analyses, QUS-defined osteoporosis status (yes/no) was defined using these T-score categories, and SOS values were retained as a continuous parameter reflecting bone quality and stiffness. Both T-scores and SOS were used to describe BMD-related characteristics and to examine the relationship between BRI and bone status.

### 2.3 Statistical analysis

All statistical analyses were performed using Python with relevant packages, including pandas,statsmodels, scipy, scikit-learn, matplotlib, and graphviz. Continuous variables were summarized as medians (interquartile range [IQR]) and compared between low- and high-BRI groups using the Mann–Whitney U test (Wilcoxon rank-sum test), whereas categorical variables were summarized as counts (percentages) and compared using Pearson’s chi-square test. BRI was examined both as a continuous variable and as a binary variable based on the optimal cutoff value derived from receiver operating characteristic (ROC) curve analysis. Bootstrap resampling with 1,000 iterations (stratified by osteoporosis status) was used to estimate areas under the ROC curves (AUCs) and 95% confidence intervals (CIs) for three nested models: Model 1 (BRI alone), Model 2 (BRI, age, and sex), and Model 3 (BRI, age, sex, SBP, TC, LDL-C, HDL-C, TG, current smoking, alcohol use, hypertension, diabetes, CHD, and stroke). BMI and WC were not entered simultaneously with BRI because these anthropometric measures share body-size information and could introduce multicollinearity or overadjustment. Their performance was therefore evaluated in separate head-to-head sensitivity analyses. Logistic regression was applied to evaluate the association between BRI and osteoporosis across these adjustment levels, with results expressed as odds ratios (ORs) and 95% CIs. The continuous BMD-related parameters did not follow a normal distribution. Robust regression with MM-estimation was therefore preferred over ordinary least-squares linear regression because it combines a high breakdown point with high statistical efficiency and is less sensitive to non-normality and influential observations, thereby providing more reliable estimates of the associations between BRI and these parameters. The primary exposure/outcome complete-case population included 1,444 participants; analyses requiring the fully adjusted covariate set used a model-specific complete-case sample of 1,408 participants. In further analyses, the discriminative performance of BRI was directly compared with that of BMI and WC using ROC analysis across the same three nested models (**Supplementary Figure 1**). Model calibration was evaluated using decile-based calibration plots, calibration-in-the-large (α), calibration slope (β), and the Hosmer–Lemeshow test. Decision curve analysis (DCA) was performed to assess net clinical benefit across threshold probabilities. At the optimal BRI cutoff of 4.5, positive predictive value (PPV) and negative predictive value (NPV) were calculated (**Supplementary Figure 2**).

## 3 Results

### 3.1 Bootstrap ROC curve analysis for BRI discriminative performance for osteoporosis

**Figure 1:**
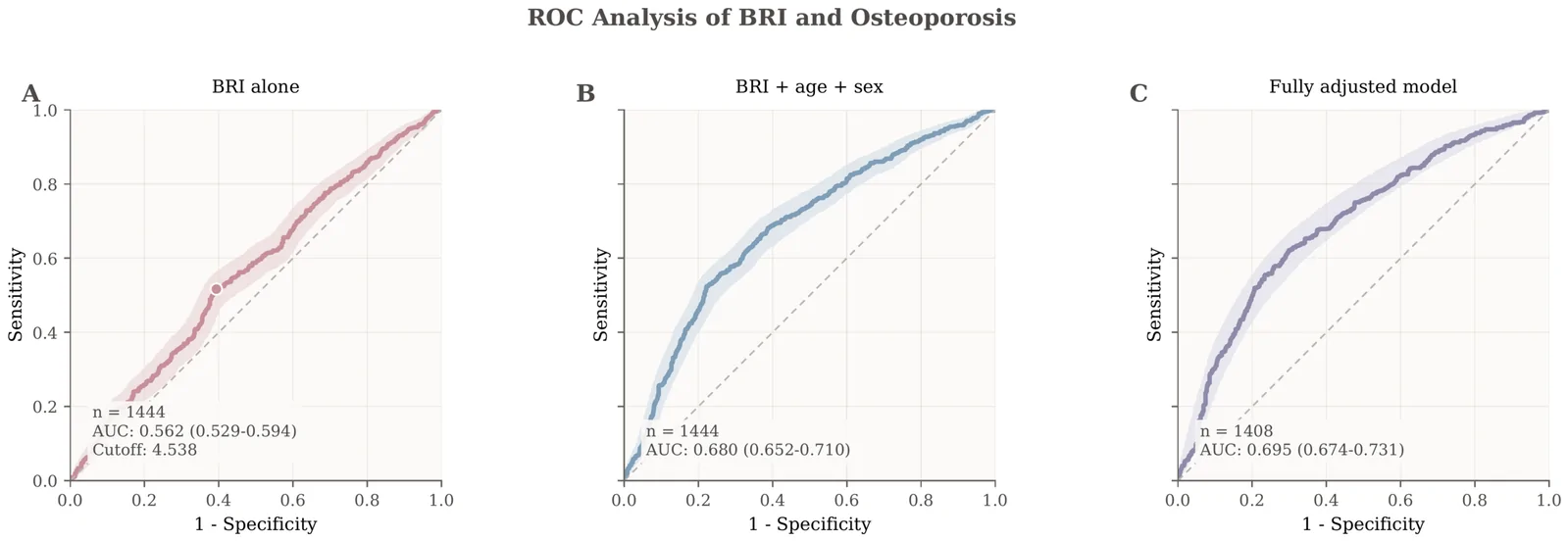
ROC curves for BRI-based QUS-defined osteoporosis models. **(A)** Model 1 includes only BRI; **(B)** Model 2 includes BRI, age, and sex; and **(C)** Model 3 additionally adjusts for lifestyle and cardiometabolic covariates, excluding BMI. AUCs with 95% CIs are shown.

Bootstrap resampling (1,000 iterations, stratified by osteoporosis status) was used to evaluate BRI discriminative performance for osteoporosis across three incremental models, with results visualized in **Figures 1A–C**. Model 1 (BRI alone) exhibited modest discriminative ability, with an AUC of 0.562 (95% CI: 0.529–0.594), and the optimal BRI cutoff determined by the Youden index was 4.538. This value was rounded to 4.5 for clinical interpretability and used to categorize participants into low (< 4.5) and high (>= 4.5) BRI groups for subsequent analyses. Adding age and sex in Model 2 improved the AUC to 0.680 (95% CI: 0.652–0.710). Further adjustment for all prespecified covariates, excluding BMI, yielded an AUC of 0.695 (95% CI: 0.674–0.731), with the Model 3 analysis based on 1,408 participants with complete covariate data.

To contextualize the discriminative performance of BRI, a direct ROC comparison with BMI and WC was conducted using the same three-model framework (**Supplementary Figure 1**). Because BMI had three additional missing values, the BRI and WC analyses included 1,444 participants in Models 1–2 and 1,408 in Model 3, whereas the BMI analyses included 1,441 and 1,405 participants, respectively. In Model 1, BRI yielded an AUC of 0.562 (95% CI: 0.529–0.594), compared with 0.525 (95% CI: 0.492–0.556) for BMI and 0.500 (95% CI: 0.468–0.530) for WC. After adjustment for age and sex, the AUCs converged (BRI: 0.680; BMI: 0.682; WC: 0.678). The fully adjusted estimates were also similar (BRI: 0.695; BMI: 0.696; WC: 0.693). Thus, BRI showed only a small unadjusted difference from BMI and WC, with no material discriminative advantage after covariate adjustment.

At the empirical BRI cutoff of 4.5, sensitivity was 52.3%, and specificity was 59.5%. Given the observed osteoporosis prevalence of 35.2%, the PPV was 41.2% (266/645), and the NPV was 69.6% (556/799). Calibration-in-the-large (α) was approximately zero, and the apparent calibration slope (β) was approximately 1.0 for each model (**Supplementary Figures 2A–C**). Hosmer–Lemeshow tests did not indicate lack of fit for Model 1 (χ² = 5.35, p = 0.720) or Model 2 (χ² = 12.29, p = 0.139), whereas Model 3 showed evidence of miscalibration (χ² = 16.33, p = 0.038). In decision curve analysis (**Supplementary Figures 2D–F**), net benefit exceeded both treat-all and treat-none strategies over threshold probability ranges of approximately 0.25–0.43 for Model 1, 0.18–0.55 for Model 2, and 0.17–0.57 for Model 3, apart from minor local fluctuations. These internal, apparent-performance estimates do not establish clinical utility.

### 3.2 Baseline characteristics by BRI category (cutoff = 4.5)

Of the 1,444 participants, 799 (55.3%) were classified as having low BRI (<4.5) and 645 (44.7%) as having high BRI (≥4.5), with significant differences in baseline characteristics between groups (**Table 1**) that aligned with established associations between body composition and health outcomes. Demographically, the high BRI group was slightly older (median, 60 vs. 59 years, P = 0.002), had a lower proportion of men (37% vs. 45%, P = 0.002), and had a lower prevalence of current smokers (19% vs. 25%, P = 0.012), whereas there was no significant difference in alcohol consumption. Chronic conditions were more prevalent in the high BRI group, including hypertension (48% vs. 35%, P = 1.94 x 10-7) and diabetes mellitus (11% vs. 7.5%, P = 0.043), whereas CHD and stroke rates were similar. Biochemical and anthropometric parameters reflected expected metabolic differences: the high BRI group had lower HDL-C, higher triglycerides and fasting glucose levels, and substantially larger waist circumference, higher BMI, and greater body weight (all P < 0.001 for key anthropometrics). Critically, osteoporosis-related measures showed worse bone health in the high BRI group, with lower median T-scores (–2.20 vs. –1.70, P = 6.96 x 10-9) and lower SOS (3774 m/s vs. 3828 m/s, P = 6.80 x 10-10), indicating an association between higher BRI and lower QUS-derived T-scores and SOS.

**Table 1:** Baseline characteristics by BRI (cutoff = 4.5).

Variable	Overall (N = 1,444)	Low BRI (<4.5) (N = 799)	High BRI (≥4.5) (N = 645)	*P*-value
Demographics				
Age, years	60 (54, 68)	59 (53, 67)	60 (55, 68)	0.002
Male sex	595 (41%)	358 (45%)	237 (37%)	0.002
Lifestyle factors				
Current smoking	323 (22%)	199 (25%)	124 (19%)	0.012
Alcohol use	129 (8.9%)	66 (8.3%)	63 (9.8%)	0.365
Chronic conditions				
Hypertension	587 (41%)	276 (35%)	311 (48%)	1.94e−07
Diabetes mellitus	129 (8.9%)	60 (7.5%)	69 (11%)	0.043
Coronary heart disease	93 (6.4%)	55 (6.9%)	38 (5.9%)	0.512
Stroke	155 (11%)	79 (9.9%)	76 (12%)	0.284
Metabolic and lipid profiles				
Total cholesterol, mmol/L	5.22 (4.22, 6.44)	5.14 (4.16, 6.43)	5.28 (4.34, 6.49)	0.129
LDL-C, mmol/L	2.95 (2.19, 4.16)	2.94 (2.16, 4.15)	2.97 (2.23, 4.17)	0.322
HDL-C, mmol/L	1.40 (1.22, 1.62)	1.43 (1.24, 1.67)	1.36 (1.18, 1.57)	3.94e−06
Triglycerides, mmol/L	1.63 (1.16, 2.27)	1.41 (1.06, 2.10)	1.83 (1.39, 2.43)	1.95e−15
Glucose, mmol/L	5.60 (5.30, 6.30)	5.60 (5.20, 6.10)	5.80 (5.30, 6.50)	2.77e−05
HbA1c, %	5.50 (5.20, 6.20)	5.50 (5.20, 6.20)	5.50 (5.20, 6.20)	0.494
Blood pressure				
SBP, mmHg	152 (139, 164)	151 (135, 163)	153 (142, 164)	0.001
DBP, mmHg	92 (82, 101)	90 (81, 101)	93 (82, 102)	0.013
Anthropometric measures				
Waist circumference, cm	88 (81, 95)	82 (77, 87)	96 (91, 101)	3.07e−171
BMI, kg/m²	25.7 (23.5, 28.1)	23.9 (22.2, 25.5)	28.2 (26.6, 29.9)	6.07e−147
Height, cm	159 (153, 166)	160 (155, 167)	158 (152, 164)	4.80e−07
Weight, kg	65 (58, 73)	62 (55, 68)	70 (64, 79)	3.23e−55
Body roundness index	4.34 (3.56, 5.31)	3.66 (3.13, 4.12)	5.43 (4.91, 6.06)	1.13e−234
Osteoporosis-related measures				
T-score	−1.90 (−3.00, −0.80)	−1.70 (−2.80, −0.50)	−2.20 (−3.20, −1.20)	6.96e−09
SOS, m/s	3801.5 (3696, 3916)	3828 (3718, 3942)	3774 (3674, 3874)	6.80e-10

Continuous variables are presented as median (interquartile range); categorical variables are presented as n (%). P-values were derived from the Mann–Whitney U test (Wilcoxon rank-sum test) for continuous variables and Pearson’s chi-square test for categorical variables.

### 3.3 Regression analyses of BRI and QUS-defined osteoporosis

Logistic regression (for osteoporosis) and robust regression (for continuous BMD parameters; T-score and SOS) were used to evaluate the association between BRI and osteoporosis across three adjustment models (**Table 2**). For continuous BRI, the OR for osteoporosis was significant in Model 1 (OR = 1.180, 95% CI: 1.085–1.283; P < 0.001), Model 2 (OR = 1.119, 95% CI: 1.026–1.221; P = 0.011), and Model 3 (OR = 1.112, 95% CI: 1.013–1.221; P = 0.026). Categorical BRI showed a similar pattern: high BRI was associated with higher odds of osteoporosis in Model 1 (OR = 1.606, 95% CI: 1.292–1.996; P < 0.001), Model 2 (OR = 1.432, 95% CI: 1.141–1.797; P = 0.002), and Model 3 (OR = 1.404, 95% CI: 1.105–1.783; P = 0.005). Robust regression linked higher BRI to lower SOS and T-score across all three models, including Model 3 for SOS (beta = –14.7546, SE = 3.3598; P < 0.001) and T-score (beta = –0.1437, SE = 0.0344; P < 0.001).

**Table 2:** Association between BRI and QUS-defined osteoporosis/BMD parameters.

Outcome/parameter	Predictor	Model 1 OR (95% CI)/β (SE)	*P*-value	Model 2 OR (95% CI)/β (SE)	*P*-value	Model 3 OR (95% CI)/β (SE)	*P*-value
Osteoporosis (logistic regression)							
	BRI (per 1-unit increase)	1.180 (1.085, 1.283)	<0.001	1.119 (1.026, 1.221)	0.011	1.112 (1.013, 1.221)	0.026
	High BRI (≥ 4.5) vs Low BRI (< 4.5)	1.606 (1.292, 1.996)	<0.001	1.432 (1.141, 1.797)	0.002	1.404 (1.105, 1.783)	0.005
BMD parameters (robust regression)							
Speed of Sound (SOS)	BRI (per 1-unit increase)	-20.7087 (3.4163)	<0.001	-15.3955 (3.1696)	<0.001	-14.7546 (3.3598)	<0.001
T-score	BRI (per 1-unit increase)	-0.1991 (0.0345)	<0.001	-0.1498 (0.0325)	<0.001	-0.1437 (0.0344)	<0.001

Model Adjustment Definitions: Model 1: Unadjusted (no covariates). Model 2: Adjusted for age and sex (key demographic confounders). Model 3: Adjusted for age, sex, SBP, TC, LDL-C, HDL-C, TG, current smoking, alcohol use, hypertension, diabetes, CHD, and stroke. Statistical methods: logistic regression, presented as odds ratio (OR) with 95% confidence interval (95% CI) for osteoporosis risk; robust regression (MM-estimation), presented as regression coefficient (beta) with standard error (SE) for continuous BMD parameters (SOS = Speed of Sound; T-score = bone mineral density T-score); negative beta indicates lower BMD with increasing BRI. BRI grouping: high BRI = BRI ≥ 4.5 (reference: low BRI = BRI < 4.5).

### 3.4 RCS analysis of BRI and QUS-defined osteoporosis risk

**Figure 2:**
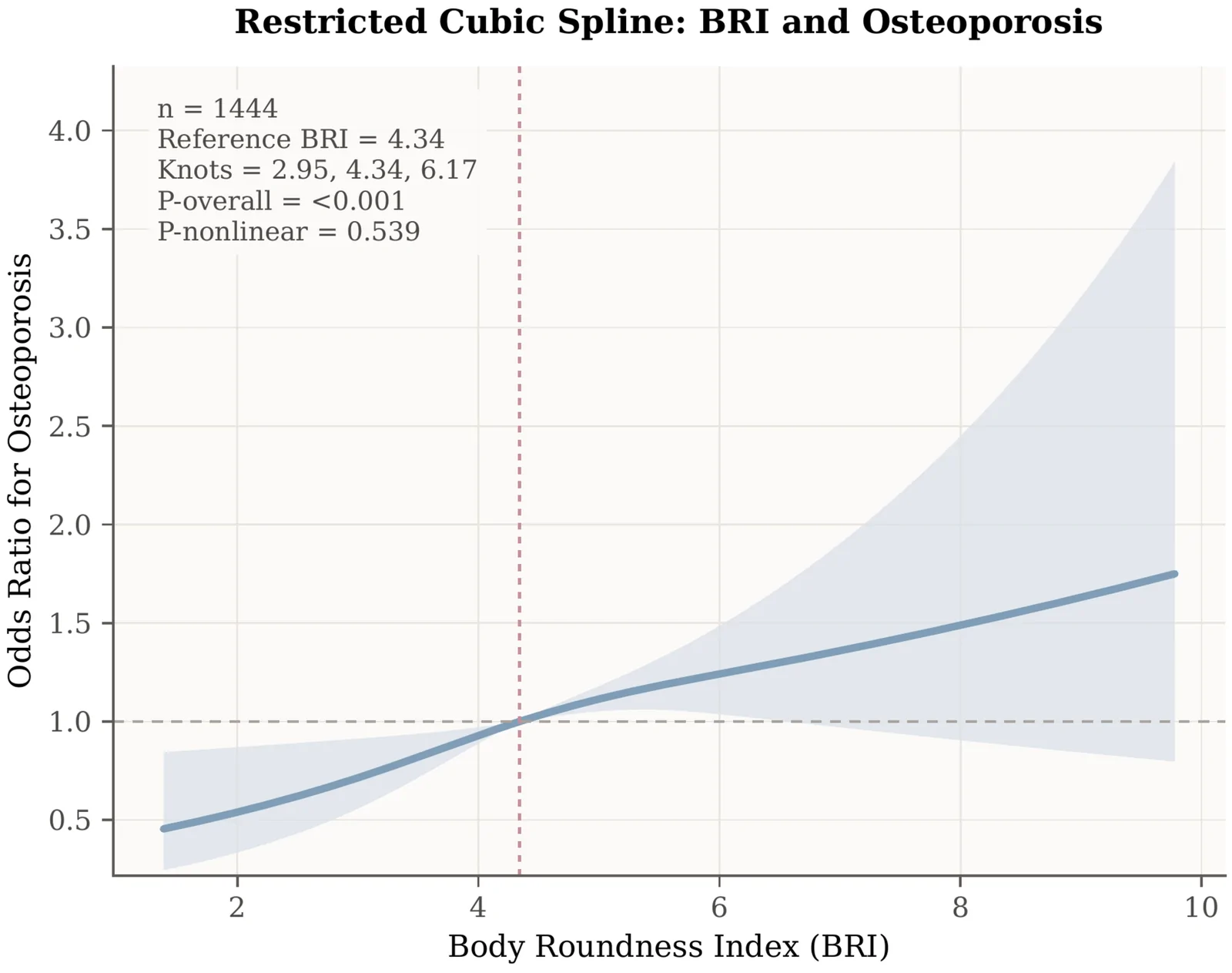
Restricted cubic spline analysis of BRI and QUS-defined osteoporosis. The spline curve depicts odds ratios for QUS-defined osteoporosis across the continuous BRI range, with 95% CIs and the median BRI used as the reference value.

RCS analysis (**Figure 2**) demonstrated an approximately linear increase in the odds of osteoporosis across the full range of BRI. The spline curve rose steadily without a pronounced inflection point, and the test for non-linearity was not statistically significant (P for non-linearity = 0.539), indicating that a linear relationship adequately characterizes the association. The overall association was statistically significant (P-overall < 0.001). The reference value was set at the median BRI (4.34), with knots placed at the 10th, 50th, and 90th percentiles (2.95, 4.34, and 6.17, respectively).

### 3.5 Association between BRI and BMD-related parameters (density scatter plots)

**Figure 3:**
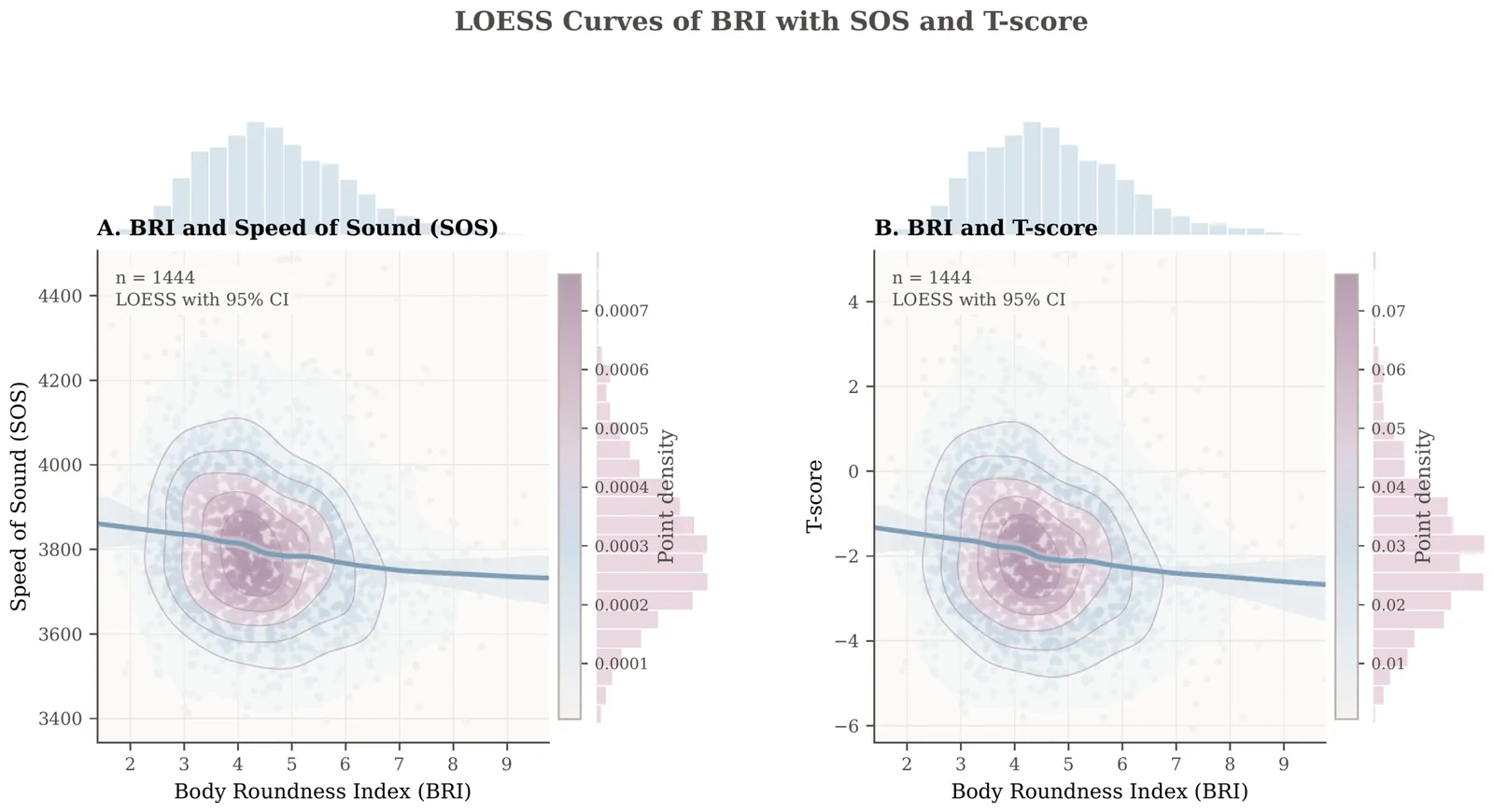
LOESS curves of BRI with SOS and T-score. Panel **(A)** shows BRI versus Speed of Sound (SOS), and panel **(B)** shows BRI versus T-score; two-dimensional density contours and LOESS curves illustrate inverse relationships between BRI and both bone-related parameters.

Density scatter plots with LOESS curves (**Figures 3A, B**) illustrated the relationship between continuous BRI and two key BMD-related parameters, SOS and T-score, showing negative trends consistent with poorer bone health at higher BRI. Panel A shows BRI versus SOS, and Panel B shows BRI versus T-score. Across 1,444 complete observations, the LOESS curves indicated that SOS and T-score tended to decline as BRI increased, consistent with the interpretation that higher BRI is associated with poorer ultrasound-based bone indicators.

### 3.6 BRI distribution according to osteoporosis status (raincloud plot)

**Figure 4:**
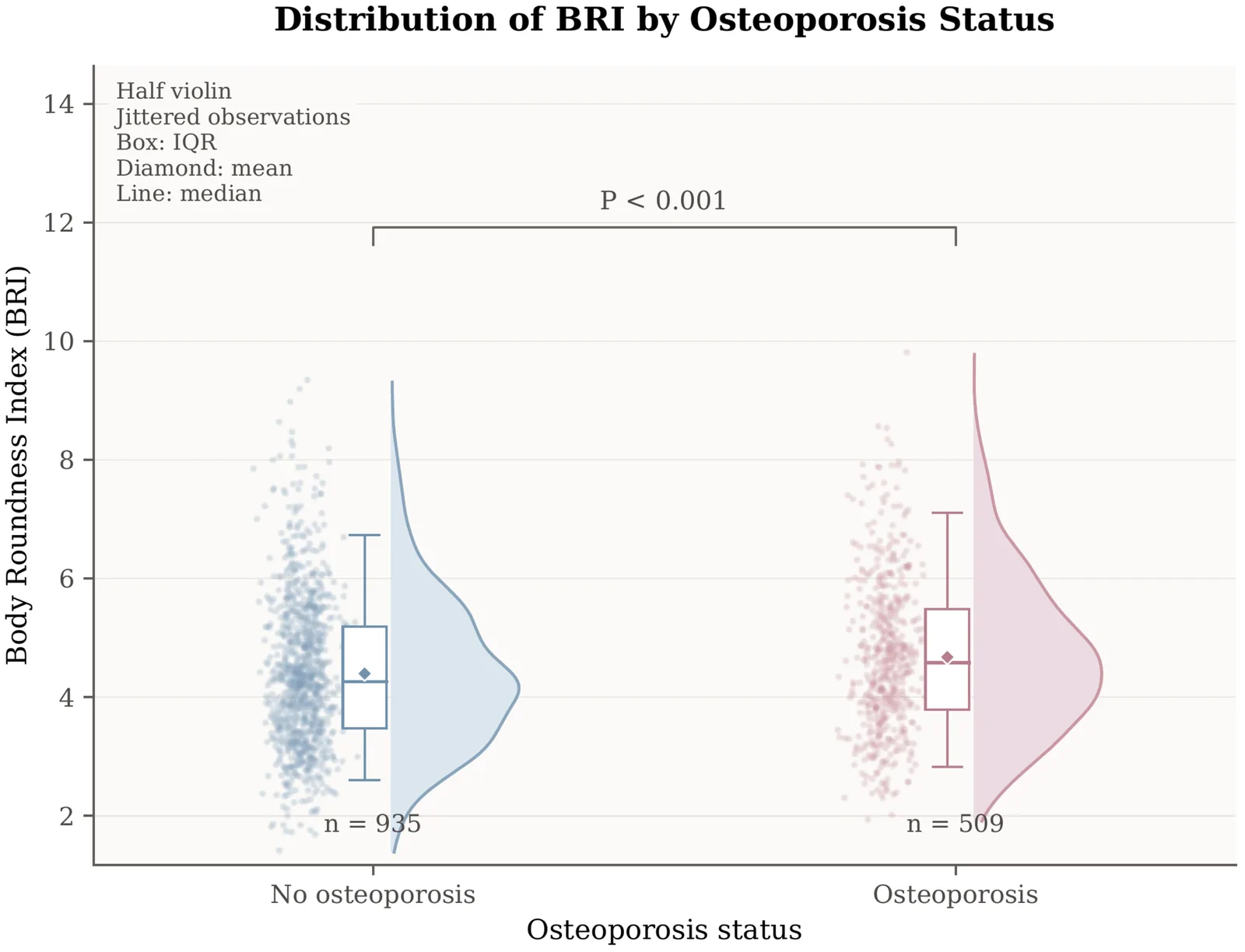
Distribution of BRI by QUS-defined osteoporosis status. Distributions of BRI are shown for participants with and without QUS-defined osteoporosis, combining half-violin density plots, jittered observations, and box summaries.

The distribution plot (**Figure 4**) illustrated clear differences in BRI distribution between individuals with and without osteoporosis, with the overall distribution for the osteoporosis group shifted to the right (Mann–Whitney U test, P < 0.001). Mean BRI was 4.68 among participants with osteoporosis and 4.40 among those without osteoporosis, while the corresponding median values were 4.58 and 4.26, respectively. The combination of half-violin density curves, jittered observations, and boxplots showed that higher BRI values were consistently observed among participants with osteoporosis.

### 3.7 Subgroup analyses (Forest plots)

**Figure 5:**
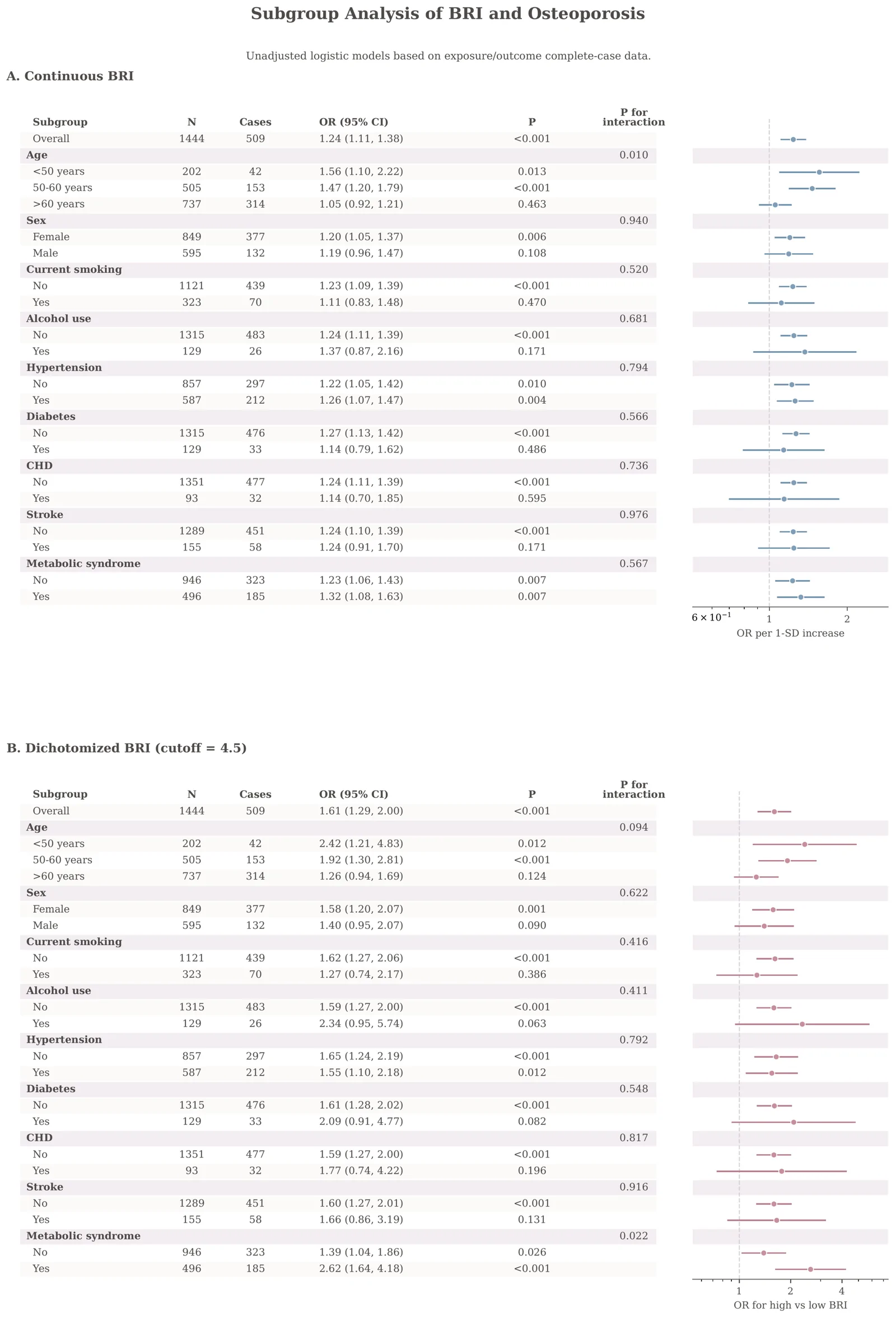
Subgroup analyses of the association between BRI and QUS-defined osteoporosis. Forest plots present subgroup-specific ORs and 95% CIs for **(A)** continuous BRI and **(B)** dichotomized BRI using a cutoff of 4.5, with P values for interaction indicating effect modification.

Forest plots (**Figures 5A, B**) evaluated the consistency of unadjusted associations between BRI and osteoporosis across subgroups, with analyses stratified by BRI as both a continuous variable (per 1-SD increase) and a categorical variable (high ≥ 4.5 vs. low < 4.5), and interaction P-values used to assess effect modification. Overall, continuous BRI was associated with higher odds of osteoporosis (OR = 1.24, 95% CI: 1.111–1.379; P < 0.001), while categorical BRI showed higher odds in the high- versus low-BRI group (OR = 1.61, 95% CI: 1.292–1.996; P < 0.001). For continuous BRI, the association varied by age group (P for interaction = 0.010), with stronger estimates among participants younger than 50 years and those aged 50–60 years than among those older than 60 years. For categorical BRI, the association was stronger among participants with metabolic syndrome (OR = 2.62, 95% CI: 1.641–4.179) than among those without metabolic syndrome (OR = 1.39, 95% CI: 1.040–1.860; P for interaction = 0.022).

## 4 Discussion

### 4.1 Summary of key findings

This cross-sectional study of 1,444 community-dwelling middle-aged and older adults systematically evaluated the association between BRI and osteoporosis, yielding five key findings. First, BRI alone showed limited discrimination for osteoporosis (AUC = 0.562, 95% CI: 0.529–0.594). Adding age and sex increased the AUC to 0.680 (95% CI: 0.652–0.710), while the fully adjusted model reached 0.695 (95% CI: 0.674–0.731). These values indicate that the majority of the discriminative information came from the covariates and that overall performance remained moderate. An empirical BRI cutoff of 4.5, derived and evaluated within this cohort, categorized 44.7% of participants as high BRI (≥4.5). High BRI was associated with higher odds of osteoporosis in unadjusted logistic regression (OR = 1.606, 95% CI: 1.292–1.996) compared with low BRI (<4.5). However, this internally optimized threshold requires external validation and should not be interpreted as a clinically established decision cutoff. Second, baseline characteristics revealed that participants with high BRI had worse bone health (lower T-scores and SOS) and a higher prevalence of metabolic comorbidities (hypertension and diabetes) and adverse lipid and glucose profiles than participants with low BRI, aligning with adiposity- and nutrition-related pathways linked to bone loss. Third, BRI showed inverse relationships with continuous bone parameters, including SOS and T-score, and these associations persisted after full covariate adjustment excluding BMI. Fourth, the association between BRI and osteoporosis appeared approximately linear across the BRI range, and subgroup analyses suggested stronger categorical associations among individuals with metabolic syndrome. Fifth, sensitivity analyses showed that the discriminative performance of BRI was comparable to that of BMI and WC after covariate adjustment, and calibration was acceptable for Models 1 and 2, whereas Model 3 showed possible miscalibration; decision curve analysis indicated modest, threshold-dependent net benefit.

### 4.2 Comparison with similar studies

Several recent studies have directly examined the relationship between BRI and bone outcomes. Analyses of US adults from NHANES and older patients undergoing comprehensive geriatric assessment have reported inverse associations between BRI and BMD, together with higher odds of osteoporosis at elevated BRI levels, although effect sizes and sex-specific patterns varied across studies (11–13). Our findings are broadly consistent with these reports by showing positive associations between higher BRI and osteoporosis across stepwise regression models using the updated covariate strategy. This extends the evidence to a community-based Chinese population assessed with tibial quantitative ultrasound and provides an empirically derived cutoff of 4.5. Traditional anthropometric measures such as BMI and WC, although widely used, have shown heterogeneous relationships with bone outcomes in previous studies, likely because they do not distinguish fat from lean mass or capture regional fat distribution (5–8). In this context, BRI may help reflect central adiposity relevant to skeletal health, although its contribution should still be interpreted alongside BMI and cardiometabolic profile. Beyond BRI, other composite adiposity indices, including A Body Shape Index (ABSI), the atherogenic index of plasma, weight-adjusted waist indices, the triglyceride-glucose index, and the Visceral Adiposity Index (VAI), have also been linked to BMD or osteoporosis in recent cross-sectional analyses (14, 17–22). A direct ROC comparison in the present cohort showed that BRI, BMI, and WC performed similarly after adjustment for age and sex (Model 2 AUCs: 0.680, 0.682, and 0.678, respectively; **Supplementary Figure 1**), suggesting that the incremental discriminative advantage of BRI over conventional measures is limited in this population. These findings extend, rather than originate, the existing evidence on BRI and bone outcomes (9–13).

### 4.3 Potential biological explanations

Several biological pathways linking visceral adiposity to bone metabolism have been proposed in prior literature. However, none were measured in this study, and they should not be interpreted as explanations established by the present analysis. First, chronic low-grade inflammation, a hallmark of excess visceral fat, disrupts the balance between bone formation and resorption. Visceral adipocytes secrete pro-inflammatory cytokines [e.g., interleukin-6 [IL-6] and tumor necrosis factor-alpha (TNF-alpha)] that inhibit osteoblast activity [via suppression of runt-related transcription factor 2 (RUNX2)] and stimulate osteoclast differentiation (via upregulation of receptor activator of nuclear factor-kappaB ligand [RANKL]) (20, 24, 25). Prior studies suggest that inflammation and insulin resistance may be greater in metabolic syndrome (7, 8, 23). However, because no inflammatory or metabolic biomarkers were measured, this literature-based hypothesis cannot explain the subgroup difference observed in the present study. Second, insulin resistance, common among individuals with high BRI, may impair bone quality by reducing osteocalcin activity (a bone-derived hormone that regulates insulin sensitivity) and disrupting calcium metabolism. Insulin-resistant states may also increase renal calcium excretion and reduce 1,25-dihydroxyvitamin D production, further compromising bone mineralization (21, 23, 30). Third, prior research suggests that adiposity may influence estrogen metabolism after menopause (2–4, 24, 25, 31). However, menopausal status and hormone replacement therapy were unavailable in this cohort, so this pathway could not be evaluated in the present analysis. Collectively, these pathways represent plausible mechanisms that may underlie the observed BRI–osteoporosis association. It should be noted that no mechanistic biomarkers were measured in this cross-sectional study. The pathways discussed above are biologically plausible but remain speculative and require validation in prospective studies incorporating biomarker mediation analyses.

### 4.4 Potential clinical implications

The clinical implications should be interpreted cautiously. BRI alone had limited discriminatory ability (AUC = 0.562), and the fully adjusted model achieved only moderate discrimination (AUC = 0.695). Although BRI is inexpensive and readily calculated from waist circumference and height (9–13, 26), these practical features do not establish clinical utility. Prior studies have also reported population-specific associations between BRI and bone outcomes (11–13), reinforcing the need for external validation. The internally derived cutoff of 4.5 should not be used as a stand-alone decision threshold. The stronger association in participants with metabolic syndrome highlights the relevance of integrated cardiometabolic and skeletal health assessments. Patients with both high BRI and metabolic syndrome may merit closer attention to lifestyle and nutritional factors, including weight management, physical activity, adequate protein and calcium intake, and anti-inflammatory dietary patterns (2–4, 6–8, 23, 29, 32, 33). The observed BRI–osteoporosis association may support risk communication regarding central adiposity and bone health. At most, BRI may provide supplementary anthropometric information within established risk-assessment pathways, but the present data do not support its use as an independent community screening test. The PPV at the 4.5 cutoff was only 41.2%, indicating that the majority of screen-positive individuals did not have QUS-defined osteoporosis. The NPV of 69.6% likewise indicates that a negative result does not reliably exclude disease. Calibration was acceptable for Models 1 and 2, whereas Model 3 showed possible miscalibration. Together with the modest DCA net benefit, these findings support positioning BRI only as an adjunctive indicator within a broader assessment framework.

### 4.5 Strengths and limitations

This study has several notable strengths. First, the large sample size and community-based recruitment enhance the generalizability of the findings to middle-aged and older adults, a group at highest risk of osteoporosis and closely linked to nutrition- and lifestyle-related risk factors (1–4). Second, we used multiple complementary statistical methods to evaluate BRI, including bootstrap ROC analysis to determine an empirical cutoff, restricted cubic spline models to clarify the dose-response relationship, robust regression for continuous BMD parameters to reduce the influence of outliers, and subgroup analyses to test for effect modification, with covariate adjustment applied to the prespecified regression and ROC models where appropriate. The BRI calculation and QUS assessment followed established anthropometric and peripheral bone-assessment approaches (9, 10, 26).

However, several limitations should be acknowledged. First, the cross-sectional design precludes causal inference; although higher BRI was associated with greater odds of osteoporosis, we cannot determine temporal ordering or exclude reverse causality. Second, BRI and osteoporosis status were each measured at a single time point, which did not allow us to examine trajectories of adiposity or bone loss over time or to capture incident fractures. Third, BMI and WC were not included with BRI in Model 3 because of their close anthropometric overlap, but this choice does not remove confounding by overall body size or composition. Lean mass, menopausal status, physical activity, dietary calcium and vitamin D intake, supplement use, systemic glucocorticoid exposure, hormone replacement therapy, other relevant medications, and fracture history were unavailable. Residual confounding may therefore be substantial, and the observed associations should be interpreted accordingly. Fourth, participants were community-dwelling adults from a single region in China who attended health examinations, thus caution is warranted when generalizing these findings to other populations with different ethnic backgrounds, healthcare systems, or osteoporosis prevalence. Fifth, osteoporosis was classified using tibial QUS rather than central DXA, the clinical reference standard for osteoporosis diagnosis. Tibial QUS assesses bone quality at a peripheral site and has only moderate agreement with hip/spine DXA (26); consequently, the present findings pertain to a QUS-derived phenotype of low bone quality rather than DXA-confirmed osteoporosis. Misclassification relative to DXA may bias the observed associations. If QUS misclassification is non-differential with respect to BRI, the effect estimates would be attenuated toward the null, whereas differential misclassification could bias estimates in either direction. Sixth, the BRI cutoff of 4.5 was derived and evaluated in the same cohort, which may lead to optimistic estimates of its performance. Its transportability remains unknown and requires validation in independent populations before any clinical application.

## 5 Conclusion

In summary, this study demonstrates that BRI is associated with QUS-defined osteoporosis in middle-aged and older adults across the updated stepwise adjustment models, with an empirically derived cutoff of 4.5 and generally consistent unadjusted subgroup patterns. BRI alone showed limited discrimination and performed similarly to BMI and WC after adjustment. It should therefore be considered only as an adjunctive indicator within a broader assessment framework, not as a stand-alone predictor or screening tool. The approximately linear association and stronger association among participants with metabolic syndrome highlight the potential relevance of visceral adiposity in bone health assessment. Future research should validate these findings in longitudinal cohorts to clarify temporality, explore the utility of BRI in diverse ethnic groups, and investigate whether changes in BRI are accompanied by changes in bone health outcomes. The empirical cutoff and potential clinical role require external validation before any application in routine assessment. Supplementary analyses confirmed similar adjusted discrimination for BRI, BMI, and WC. Calibration findings were acceptable for Models 1 and 2, but the Model 3 Hosmer–Lemeshow result indicated possible miscalibration.

## Data Availability

The data that support the findings of this study are available from the corresponding author Haoran Wang (d201278406@alumni.hust.edu.cn), upon reasonable request. Requests to access these datasets should be directed to Haoran Wang, d201278406@alumni.hust.edu.cn.

## Supplementary material

Supplementary Figure 1 ROC Comparison of BRI, BMI, and Waist Circumference for QUS-defined Osteoporosis. Panels **(A–C)** compare the discriminative performance of BRI, BMI, and waist circumference under the unadjusted, age- and sex-adjusted, and fully adjusted model specifications, respectively. ROC curves, bootstrap-derived 95% CIs, AUCs with 95% CIs, and model-specific sample sizes are shown. The fully adjusted models additionally include SBP, TC, LDL-C, HDL-C, TG, current smoking, alcohol use, hypertension, diabetes, CHD, and stroke.

Supplementary Figure 2 Calibration and Decision Curve Analysis of BRI-Based Models for QUS-defined Osteoporosis. Panels **(A–C)** show decile-based calibration plots for Model 1 (BRI alone), Model 2 (BRI, age, and sex), and Model 3 (the fully adjusted model), respectively. Calibration-in-the-large (α), calibration slope (β), and Hosmer–Lemeshow χ² statistics with P values are reported. Panels **(D–F)** show the corresponding decision curves, comparing each model with the treat-all and treat-none strategies across threshold probabilities. Performance at the empirical BRI cutoff of 4.5 is summarized using prevalence, sensitivity, specificity, positive predictive value, negative predictive value, and the numbers of true-positive, false-positive, false-negative, and true-negative classifications.
